# Nitrogen management in rainfed castor: a comprehensive analysis of productivity, nutrient use efficiency, economics, energy flow, and carbon footprint

**DOI:** 10.3389/fpls.2026.1969600

**Published:** 2026-10-28

**Authors:** Revappa Mohan Kumar, Yamanura Madival, Venkatesh Paramesha, Ganiga Jadesha, Gopalaswamy Ranganath

**Affiliations:** 1 University of Agricultural Sciences, Bangalore, India; 2 ICAR - Central Coastal Agricultural Research Institute, Ella, India

**Keywords:** foliar nutrition, nano urea, nitrogen balance intensity, nutrient uptake, oil content, partial factor productivity, physiological efficiency

## Abstract

**Introduction:**

Nitrogen (N), an essential plant nutrient characterized by the lowest use efficiency and multiple loss pathways, challenges its management in rainfed castor. Therefore, a field experiment was conducted over three consecutive *Kharif* seasons (2022-2024) to develop an adoptable N management strategy for rainfed castor cultivation.

**Methodology:**

The study investigated the impact of eight N management modules *viz.*, Recommended rate of fertilizer (RRF) (40-40–25 kg N-P_2_O_5_-K_2_O ha^-1^), 50% recommended rate of basal N (RRBN) +2 ml l^-1^ Nano Urea at 30 and 60 days after sowing (DAS), 50% RRBN + 2% Urea spray at 30 and 60 DAS, 75% RRBN + 2 ml l^-1^ Nano Urea at 30 and 60 DAS, 75% RRBN + 2% Urea spray at 30 and 60 DAS, 100% RRBN + 2 ml l^-1^ Nano Urea at 30 and 60 DAS, 100% RRBN + 2% Urea spray at 30 and 60 DAS, and recommended rate of phosphorous and potassium (RRPK) (00-40–25 kg N-P_2_O_5_-K_2_O ha^-1^) and were arranged in randomized complete block design with three replications.

**Results and discussions:**

The study found that the combined soil and foliar application of N had a promising effect on crop productivity than soil-only N application. Aqueous solution of urea (2%) proved effective than Nano-Urea (2 ml L^-1^) for foliar fertilization of N. Specifically, application of 100% RRBN through soil (20 kg ha^-1^), followed by foliar sprays of 2% Urea at 30 and 60 DAS, improved growth and yield traits of castor. Consequently, it produced significantly higher seed yield (1546 kg ha^−1^), oil content (42.7%), and N uptake in castor (34.9 kg ha^−1^). Due to inverse relationship, N substituted via foliar fertilization enhanced the partial factor productivity (67.8–106 kg kg^-1^) compared to soil-only N application (32.5 kg kg^-1^). Foliar fertilization depletes the soil N pool as observed through +ve N balance index (10.7-12.1). Furthermore, 100% RRBN + foliar sprays of 2% Urea at 30 and 60 DAS found to be remunerative, besides enhancing energy (58,726 MJ ha^−1^), carbon (C) output (1,947 kg-CE ha^−1^), net energy and C-gain. These findings highlight that partial N substitution through foliar applications optimizes castor productivity, economics, energy, and environmental security in rainfed castor.

## 1 Introduction

Castor is an important non-edible oilseed crop grown in India. Its seeds contain 35-50% oil, rich in triglycerides made up of high-quality ricinoleic acid (90%), as well as linoleic acid, linolenic acid, oleic acid, stearic acid, and palmitic acid (McKeon, 2016). Because of its unique makeup, castor oil is an essential raw material across various industries, including pharmaceutical, cosmetic, dyeing, coating, and high-quality lubricants (Ogunniyi, 2006). India accounts for a major share of the world’s castor production (~80%). It is currently cultivated on an area of 8.28 lakh hectares, with an average productivity of 1.92 tons per hectare; approximately 15.88 lakh tons of castor seeds are produced annually in India (ICAR-IIOR, 2025). From the study conducted by ICAR-IIOR (2025) it was observed that the productivity of castor is significantly higher (~2.2 t ha^-1^) in irrigated conditions of north-western India (Gujarat, Rajasthan, and Haryana) compared to rainfed regions of southern India (~0.5 t ha^-1^). Despite its lower productivity, it occupies a substantial area in the climatically fragile rainfed agroecosystems of southern India.

In southern India, castor is mainly cultivated during the rainy season, characterized by erratic rainfall with extended dry spells. This makes N management particularly challenging. In marginal soils under rainfed conditions, the crop often suffers from frequent moisture stress and improper nutrition; as a result, farmers fail to achieve their potential yield (Kumar and Yamanura, 2019). Several studies have clearly shown that N stress is the most yield-limiting factor, causing severe (20-50%) yield loss (Calcagno et al., 2023; Patel et al., 2023; Gato et al., 2024). While N is required in higher rates than other mineral nutrients for optimal growth and development of castor (Xue et al., 2017). For instance, a study conducted by Reddy and Matcha (2010) highlighted that N is vital in improving growth, yield, and oil content in castor. Similarly, in rainfed *Vertisols* of Andhra Pradesh, India Rani et al. (2014) found significant gains in castor growth and productivity with higher N rates (80 kg ha^-1^) compared to lower rates (0, 40, and 60 kg ha^-1^). Likewise, Xue et al. (2017) from China also revealed that applying higher N fertilizer rates significantly boosts castor seed yield, with a linear increase observed up to 300 kg N per hectare. Under irrigated conditions of Gujarat Patel et al. (2023)also observed higher rates of N (180 kg ha^-1^) consistently improved the major growth and yield traits of castor compared to lower N levels. In another study, Calcagno et al. (2023), working on typical *xerofluvent* soils in Italy, found that applying 120 kg N per hectare resulted in the highest seed yield compared to 60 kg N per hectare, confirming the importance of adequate N levels for maximizing productivity.

The N management in castor is largely covered by soil application of chemical fertilizers. Alongside, urea remains the predominant source of N fertilizer in India; its use efficiency in optimal field conditions rarely exceeds 30% (Duan et al., 2016). To enhance the use efficiency of N fertilizer vis-à-vis optimal crop nutrition in castor, there is an urgent need to revisit N management. In this context, integrated use of soil and foliar modes of nutrient management appears to be a promising solution. Multiple studies in the recent past have emphasized the benefits of soil-cum-foliar nutrient management practices in different crops (Sadeghi-Bakhtavari and Hazrat, 2021; Kumar et al., 2024; Gopinath et al., 2025). In addition to soil application, foliar fertilization helps in partial substitution of soil-applied fertilizers and also in mitigating nutrient losses caused by runoff and leaching while enhancing nutrient availability to the crop plants (Dass et al., 2022). Foliar application of nutrients, particularly alleviating water stress during drought periods and imparting balanced nutrition in rainfed cropping (Sadeghi-Bakhtavari and Hazrat, 2021). In addition to these advantages, nutrient substitution through foliar means helps in optimization of energy metrics and C footprints in field crop production by way of improving use efficiency and mitigating nutrient losses (Kumar et al., 2024). Based on the comprehensive review, N management practices in rainfed castor are limited to productivity and economic analysis. But in today’s intensive agriculture, recommendations for key crop production technologies should also focus on use-efficiency, energy matrices, and carbon footprints. This comprehensive approach helps in devising climate resilient sustainable agriculture technologies (Paramesha et al., 2025; Wang et al., 2015; Chaudhary et al., 2017; Parihar et al., 2018; Kumar et al., 2024, 2025).

Therefore, this study aims to provide a comprehensive assessment of crop performance, economics, energy analysis, and C auditing in rainfed castor. We hypothesize that the integrated use of soil and foliar N sources will improve yields, nutrient use efficiency, and optimize energy metrics and carbon footprint in rainfed castor. The objectives of this research were (1) to evaluate the effects of different N management strategies on growth, yield, economics, and oil content in castor, (2) to analyze different plant and soil based N use efficiencies indicatives for tested N management strategies, (3) to determine the energy efficiencies of N management strategies in rainfed castor, and (4) to understand the C footprint dynamics associated with different N management strategies. The results from the field experiment will aid in developing efficient N management programs that enhance productivity, profitability, energy use, and carbon footprint.

## 2 Materials and methods

### 2.1 Study site

A field experiment was conducted over three consecutive *kharif* seasons (2022-2024) at the All India Coordinated Research Project on Castor, Zonal Agricultural Research Station, University of Agricultural Sciences, Bangalore, Karnataka (India). The experimental site is situated between latitudes 13.0781° N and longitudes 77.5792° E, at an elevation of 930 meters above mean sea level. The location has distinct rainy and dry seasons due to its tropical savanna climate. During the cropping seasons (May-February), a total of 1491, 712, and 1439 mm of rainfall was received in 64, 44, and 64 rainy days of 2022-23, 2023-24, and 2024-25, respectively (**Figure 1**). Before starting the experiment in 2022, a composite soil sample was taken from the 0–15 cm depth of the soil profile and were analyzed for various soil quality parameters. The soil of the experimental site is a typical rainfed *Alfisol* with a sandy clay loam texture (USDA soil textural classification). It has a moderate level of soil organic C (0.56%), moderately acidic soil reaction (pH 6.04), low in electrical conductivity (0.11 dS m^-1^), medium in available N (366 kg ha^-1^), phosphorus (28 kg ha^-1^), and high in available potassium (434 kg ha^-1^).

**Figure 1:**
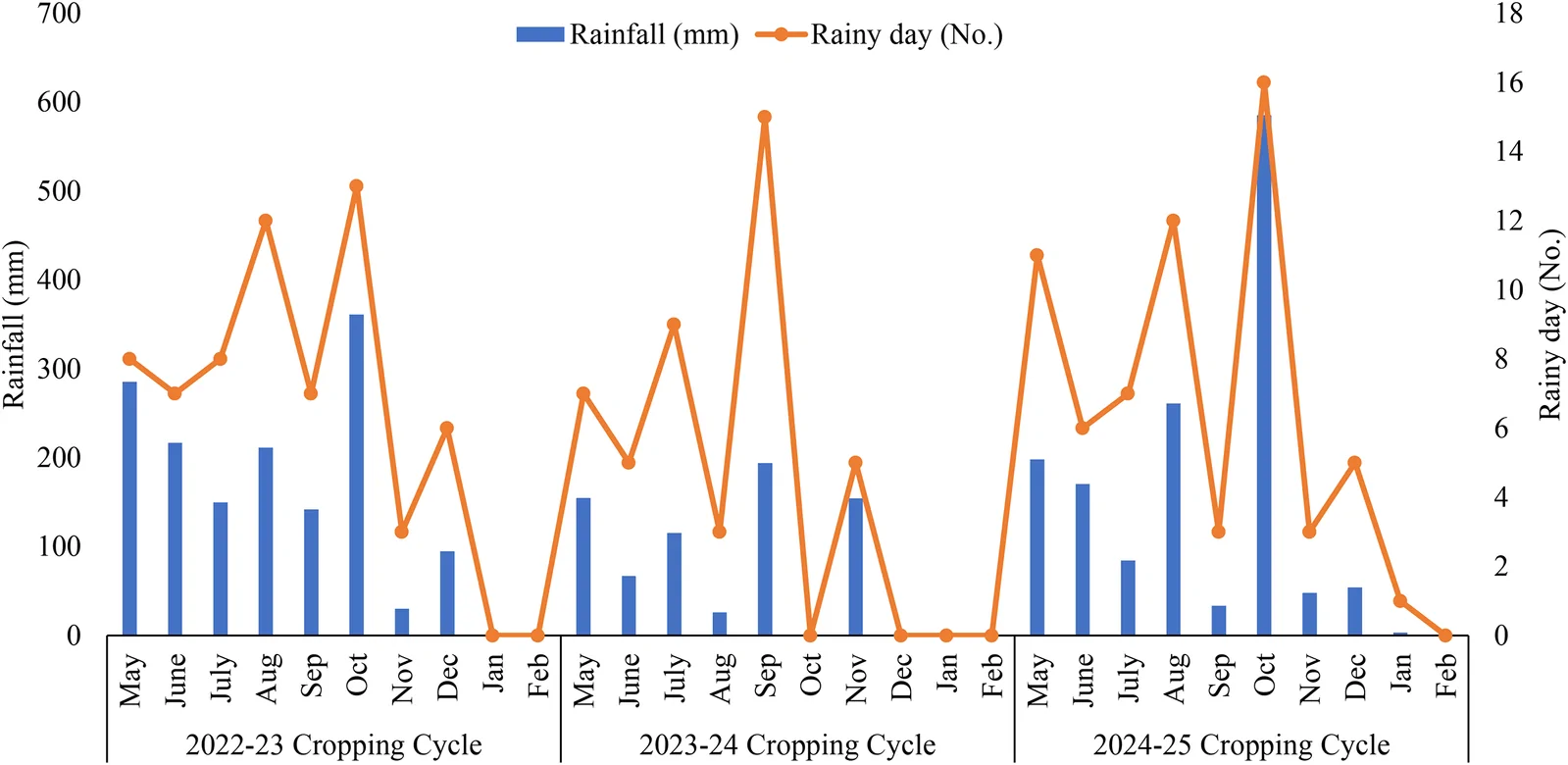
Monthly total rainfall and rainy days during the crop growth period (May 2022-Feb 2025).

### 2.2 Treatments and experimental design

The experiment comprised 8 N management modules *Viz*., Recommended rate of fertilizer (T_1_) [Recommendation of the fertilizer rate for commercial cultivation of rainfed as per the University of Agricultural Sciences, Bangalore, Karnataka package of practice where in 50% N (20 kg ha^-1^) and full dose of P_2_O_5_ (40 kg ha^-1^) and K_2_O (25 kg ha^-1^) are applied at the time of sowing, remain N is applied in two split doses at 30 and 60 DAS (RRF (40-40–25 kg N-P_2_O_5_-K_2_O ha^-1^)], 50% RRBN - + 2 ml l^-1^ Nano urea at 30 and 60 DAS (T_2_), 50% RRBN + 2% Urea spray at 30 and 60 DAS (T_3_), 75% RRBN + 2 ml l^-1^ Nano urea at 30 and 60 DAS (T_4_), 75% RRBN + 2% Urea spray at 30 and 60 DAS (T_5_), 100% RRBN + 2 ml l^-1^ Nano urea at 30 and 60 DAS (T_6_), 100% RRBN + 2% Urea spray at 30 and 60 DAS (T_7_), and 100% RRPK (00-40–25 kg N-P_2_O_5_-K_2_O ha^-1^) (T_8_). The details of the fertilizer quantity used and their application schedule are summarized in **Supplementary Table 1**. These treatments were arranged in a randomized complete block design with three identical replications. To create a good seedbed, the experimental site was ploughed with a tractor-drawn moldboard plough, followed by a cultivator, which was passed to bring the soil into fine tilth. Subsequently, the experimental area was divided into three blocks (replications), which were perpendicular to the soil fertility gradient. In each replication, eight plots were demarcated with an individual plot measuring 32.4 m^2^ (5.4 ^m^ x 6.0 m) in a sequence. Then the plots were separated from one another by raising a semi-permanent earthen bund around the plot.

In each plot, well-decomposed farmyard manure (FYM) was incorporated at a rate of 4 t ha^-1^. Then, a shallow row was opened every 0.9 m, and a fertilizer mixture containing N, P_2_O_5_, and K_2_O was applied according to the treatments at the seed drilling points, which were spaced 0.6 m apart, and mixed into the soil. The details of the fertilization schedule are provided in **Supplementary Table 1**. The hybrid castor ‘ICH-66’ was sown on 2022-06-14, 2023-06-10, and 2024-06-17. For foliar nutrition, locally available Prilled Urea (46% N w/w) and a commercial Nano Urea formulation (4% N w/v) developed by the Indian Farmers Fertilizer Cooperative Limited, India, were used. To prepare a 2% urea solution, 20 g of urea granules were dissolved in 1000 mL of water. Depending on the treatment, different concentrations of nano urea and prilled urea solutions were sprayed on the leaves of the castor plants at 30 and 60 DAS. A spry solution of 100 and 200 L ha^-1^, respectively was used during 30 and 60 DAS.

### 2.3 Growth and yield measurements

The important growth attributes, such as plant height, SPAD chlorophyll reading, number of nodes up to the primary spike, and total dry matter production (TDMP), were recorded at various crop growth stages. The SPAD chlorophyll reading was taken on the three fully opened leaves from the top of the plant/branches at 20, 40, and 70 DAS, and the mean SPAD value was calculated. The nodes were counted on the main stem in five representative tagged plants in the sampling zone up to the primary spike, and the average node number was calculated. For leaf area assessment, three plants per treatment were randomly selected at 50 and 100 DAS. The plants were carefully cut close to the ground, and the leaves were separated. The separated leaves were then passed through a LI-COR Leaf Area Meter (LI-COR Inc., Nebraska, USA) to quantify the leaf area per plant (cm^2^). Subsequently, the Leaf Area Index (LAI) was calculated using the method described by Watson (1952), applying the following Equation 1:

(1) $$LAI=\frac{Leaf\;area\left(m^{2}\right)}{Land\;area\left(m^{2}\right)}$$

For dry weight, the plants harvested for estimation of leaf area were dried under shade for 72 hours (stems, leaves, and spikes). The plant parts were transferred to a hot air oven and dried at a temperature of 64 °C until the sample reached a constant weight. Next, treatment-wise dried samples were weighed, and the total weight was measured using a weighing balance. The weight of the dried plant samples was recorded as TDMP and expressed in g plant^-1^.

Similarly, yield attributes were recorded on five randomly selected and tagged plants. The spike length was measured from the base to its tip and expressed in cm. Spike with more than 30 cm in length and the densest capsules was considered effective spikes. The effective spikes per plant were counted at each harvest. Further, capsules on the primary spike were counted and recorded as the number of capsules per primary spike. The spikes from the net plot area of each treatment were harvested separately at 120, 150, and 180 DAS. Once the capsule attained brittleness after sun drying, treatment-wise capsules were fed to the small-scale electric-operated castor decorticator. The seeds were separated from the capsules and allowed to sun-dry to attain 12-15% moisture. Seeds obtained after decortication were measured and recorded as yield per net plot area. The seed yield of each treatment in net plots was summed and converted into a hectare basis and expressed as yield in kg ha^-1^. The weight of the 100 seeds from individual treatments was randomly selected and weighed to get the 100 seed weight, and expressed in g. Soon after the harvest of all the spikes at 180 DAS, the plants within the net plot area were cut at ground level and sun-dried for 10 days. Subsequently, the dried plants were cut close to the ground, bundled, and their weight was recorded to determine the stalk yield. The biological yield was then computed by summing the seed yield and stalk yield from the net plot area, with the final value expressed in kg ha^-1^.

### 2.4 Soil and plant analysis

The textural class of the soil was determined using the international pipette method as described by Piper (1966). Soil pH was measured potentiometrically (Piper, 1966), while electrical conductivity was assessed using a conductivity bridge, following the procedure outlined by Jackson (1973). SOC content was estimated through the Walkley and Black wet oxidation method (Jackson, 1973). Available N was analyzed using the alkaline permanganate method developed by Subbaiah and Asija (1956). Available phosphorus was determined using Bray’s method, and available potassium was measured with a flame photometer, both following the protocols described by Jackson (1973).

The plants used for dry matter estimation were ground using a Willey mill and employed for analysis. The micro-Kjeldahl method was employed to assess the N content in the plant material, following Piper (1966). To prepare di-acid extracts, the method described by Jackson (1973) was utilized, employing a combination of nitric acid (HNO_3_) and perchloric acid (HClO_4_) in a 9:4 ratio. Before extraction, the samples underwent pre-digestion using 10 mL of HNO_3_ for each gram of plant material. The di-acid extract was subsequently utilized for measuring Phosphorus and Potassium. The phosphorus content was determined spectrophotometrically using the vanadomolybdate phosphoric acid yellow color method, adhering to the procedures of Jackson (1973) and Rangaiah et al. (2024). Potassium concentrations were measured utilizing a flame photometer, following the technique outlined by Jackson (1973). Based on the chemical analysis data, the absorption of each nutrient was determined as indicated by Equation 2:

(2) $$Nutrient\;uptake\left(kg\;ha^{-1}\right)=\frac{Nutrient\;content\left(\%\right)\times Dry\;weight\left(kg\;ha^{-1}\right)}{100}$$

### 2.5 Oil content estimation

The seed samples drawn from the net plot yield were subjected to oil content estimation. The oil content in the seeds of castor was determined using Nuclear Magnetic Resonance Spectroscopy (NMRS) as per the procedure described by Yadav and Murthy (2016). Subsequently, oil yield was calculated by multiplying the oil content (%) by the corresponding seed yield and dividing the product by 100 (Equation 3), by the method proposed by Raja et al. (2007).

(3) $$Oil\;yield\left(kg\;ha^{-1}\right)=\frac{Seed\;yield\left(kg\;ha^{-1}\right)\times Oil\;Content\left(\%\right)}{100}$$

### 2.6 Nitrogen efficiency indices

#### 2.6.1 Partial factor productivity

The PFP of N for each treatment was calculated as the ratio of castor seed yield to the total quantity of fertilizer N applied through soil and foliar application, as specified for the respective treatment. The PFP was estimated following the methodology proposed by Dobermann (2007) as given below (Equation 4).

(4) $$PFP\left(kg\;kg^{-1}\right)=\frac{Seed\;yield\left(kg\;ha^{-1}\right)}{Fertilizer\;N\;applied\left(kg\;ha^{-1}\right)}$$

#### 2.6.2 Nitrogen balance intensity

The NBI was calculated by subtracting total N outputs (crop removal) from N inputs (fertilizer) as suggested by IPNI (2014) through the following formula (Equation 5).

(5) $$\text{NBI}\left(\text{kg }N\;ha^{-1}\right)=N\;uptake\left(kg\;ha^{-1}\right)-N\;input\left(kg\;ha^{-1}\right)$$

#### 2.6.3 Nitrogen use efficiency of crop

The NUE_Crop_ indicates the ratio of N harvested by the crop relative to the total N inputs supplied to the crop. The values greater than 1 indicates net N removal from the soil, and less than 1 indicates N surplus in the soil as suggested by Martinez-Feria et al. (2018) and was calculated using the following formula (Equation 6).

(6) $$NUE_{Crop}=\frac{Crop\;N\;uptake\;\left(kg\;ha^{-1}\right)}{Fertilizer\;N\;applied\left(kg\;ha^{-1}\right)}$$

#### 2.6.4 Agronomic efficiency

AE of N was calculated as the increase in crop yield per unit of applied N fertilizer, as suggested by Dobermann (2007), through the following formula (Equation 7).

(7) $$AE\left(kg\;kg^{-1}\right)=\frac{Yield\;in\;N\;applied\;plot\left(kg\;ha^{-1}\right)-Yield\;in\;control\;plot\left(kg\;ha^{-1}\right)}{Fertilizer\;N\;applied\left(kg\;ha^{-1}\right)}$$

#### 2.6.5 Fertilizer-N recovery efficiency

The F-NRE was calculated as the proportion of applied N fertilizer taken up by the crop, as suggested by Dobermann (2007), through the following formula (Equation 8).

(8) $$FNRE\left(\%\right)=\frac{Plant\;N\;uptake\;in\;fertilized\;plot\left(kg\;ha^{-1}\right)-Plant\;N\;uptake\;in\;control\;plot\left(kg\;ha^{-1}\right)}{Fertilizer\;N\;applied\;\left(kg\;ha^{-1}\right)}\times 100$$

#### 2.6.6 Physiological efficiency

The PE of N was calculated to assess the crop’s ability to convert absorbed N into economic yield, as suggested by Dobermann (2007), through the following formula (Equation 9).

(9) $$PE\left(kg\;kg^{-1}\right)=\frac{Yield\;in\;N\;applied\;plot\left(kg\;ha^{-1}\right)-Yield\;in\;contol\;plot\left(kg\;ha^{-1}\right)}{Plant\;N\;uptake\;in\;fertilized\;plot\left(kg\;ha^{-1}\right)-Plant\;N\;uptake\;in\;control\;plot\left(kg\;ha^{-1}\right)}$$

#### 2.6.7 Nitrogen utilization efficiency

The NUtE was calculated to quantify the crop’s efficiency in converting absorbed N into yield (Equation 10). NUtE was determined using the formula of Moll et al. (1982).

(10) $$NUtE=\frac{Yield\;\left(kg\;ha^{-1}\right)}{Plant\;N\;uptake\;\left(kg\;ha^{-1}\right)}$$

#### 2.6.8 Nitrogen use efficiency of soil

The NUE_Soil_ refers to the efficiency with which soil-supplied N is utilized by crops for biomass production (Equation 11). It is calculated as per the method suggested by Moll et al. (1982) by using the following formula.

(11) $$NUE_{Soil}=\frac{Biological\;yield\;\left(kg\;ha^{-1}\right)}{Fertilizer\;N\left(kg\;ha^{-1}\right)+Soil\;N\left(kg\;ha^{-1}\right)}$$

#### 2.6.9 Nitrogen uptake efficiency

The NUpE quantifies the crop’s ability to absorb N from applied fertilizers. It was calculated using the following formula (Equation 12):

(12) $$NUpE\left(\%\right)=\frac{Plant\;N\;uptake\;\left(kg\;ha^{-1}\right)}{Fertilizer\;N\left(kg\;ha^{-1}\right)+Soil\;N\left(kg\;ha^{-1}\right)}\times 100$$

#### 2.6.10 Nitrogen use efficiency of yield

The NUE_Yield_ assesses the contribution of N supplied from the soil that is allocated to the yield. It was calculated as per the methodology suggested by Novoa and Loomis (1981) by using the following formula (Equation 13).

(13) $$NUE_{Yield}=NUpE\times NUtE$$

### 2.7 Economic analysis

The cost of different N management modules was calculated by conducting a detailed inventory of inputs used and processes carried out during the study on a hectare basis. The costs of each input and activity were calculated by considering the market prices of the respective years of experimentation (₹ 1.00 is equal to 85.56 $). Subsequently, gross returns, net returns, and the benefit-cost ratio were calculated by using the following formula (Equations 14–17).

(14) $$Cost\;of\;cultivation\;\left(\$ha^{-1}\right)=Inputcost\;\left(\$\right)+Labour\;cost\;\left(\$\right)$$

(15) $$Gross\;return\;\left(\$ha^{-1}\right)=Seed\;yield\;\left(kg\;ha^{-1}\right)\times Market\;price\;\left(\$kg^{-1}\right)$$

(16) $$Net\;return\;\left(\$ha^{-1}\right)=Gross\;return\;\left(\$ha^{-1}\right)-Cost\;of\;cultivation\;\left(\$ha^{-1}\right)$$

(17) $$Benefit–cost\;ratio=\frac{Gross\;return\;\left(\$\right)}{Cost\;of\;cultivation\;\left(\$\right)}$$

### 2.8 Energy calculation

To calculate the energy metrics of N management in castor, an energy inventory was prepared for the different treatments. In each N management module, inputs and operations were transformed into energy units *via* energy coefficients obtained from published literature (**Supplementary Table 2**). The input energy was calculated by summing the product of the input and process with their corresponding energy coefficients. Similarly, the energy output was determined by multiplying the seed and stalk weights by the corresponding energy coefficients, as suggested by Mandal et al. (2002). Subsequently, various energy parameters were calculated using the methodology suggested by Mittal and Dhawan (1988), as outlined in the following (Equations 18–27).

(18) $$Energy\;use\;efficiency=\frac{Energy\;output\;\left(MJ\right)}{Energy\;input\;\left(MJ\right)}$$

(19) $$Net\;energy\;gain\;\left(MJ\;ha^{-1}\right)=Total\;energy\;output\;\left(MJ\;ha^{-1}\right)-Total\;energy\;input\;\left(MJ\;ha^{-1}\right)$$

(20) $$Energy\;profitability=\frac{Net\;energy\;gain\;\left(MJ\;ha^{-1}\right)}{Total\;energy\;input\;\left(MJ\;ha^{-1}\right)}$$

(21) $$Energy\;productivity\;(kg\;MJ^{-1}=\frac{Biological\;productivity\;\left(kg\;ha^{-1}\right)}{Total\;energy\;input\;\left(MJ\;ha^{-1}\right)}$$

(22) $$Direct\;energy\;\left(MJ\;ha^{-1}\right)=Labour+Fuel+Electricity$$

(23) $$Indirect\;energy\;\left(MJ\;ha^{-1}\right)=Seeds+Fertilizer+Chemicals+Machineries$$

(24) $$Renewable\;energy\;\left(MJ\;ha^{-1}\right)=Labour+Organic\;Manure+Water\;for\;spray$$

(25) $$Non-renewable\;energy\;\left(MJ\;ha^{-1}\right)=Fuel+Elecricity+Seed+Fertilizers+Chemicals+Machinery$$

(26) $$Specific\;energy\;\left(MJ\;kg^{-1}\right)=\frac{Total\;energy\;input\;\left(MJ\;ha^{-1}\right)}{Biological\;productivity\;\left(kg\;ha^{-1}\right)}$$

(27) $$Nutrient\;energy\;ratio=\frac{Total\;energy\;output\;\left(MJ\;ha^{-1}\right)}{Total\;nutrient\;energy\;input\;\left(kg\;ha^{-1}\right)}$$

### 2.9 Carbon auditing

The C footprint of the different N management modules was calculated as per the assumption of Cradle-to-farm-gate life cycle assessment. The system boundary of this study C emission from inputs, on-farm activities, and transportation up to the point it leaves the farm were considered. The C input was derived by converting CO_2_ released from various inputs by using the C emission factor available from the published research (**Supplementary Table 3**). The C input was determined by multiplying the unit input by its specific C coefficients and expressed in terms of C equivalent per unit area and time (kg CE ha^-1^ year^-1^). The total C input in each treatment was calculated by summing the CE of different inputs. Similarly, total C output in different treatments was calculated by multiplying the biological yield by the corresponding C emission coefficient as per the methodology suggested by Lal (2004). Furthermore, using C input and output, the following C indices were determined according to the methodology suggested by Lal (2004) by using Equations 28–33.

(28) $$Carbon\;footprinting\;\left(kg\;CE\;kg^{-1}\right)=\frac{Carbon\;emission\;\left(kg\;CE\;ha^{-1}\right)}{Castor\;seed\;yield\;\left(kg\;ha^{-1}\right)}$$

(29) $$Carbon\;output\left(kg\;CE\;ha^{-1}\right)=Total\;biomass\left(Economic\;yield+biproduct\;yield\right)\;x\;0.44$$

(30) $$Net\;carbon\;gain\;\left(kg\;CE\;ha^{-1}\right)=Carbon\;output\;\left(kg\;CE\;ha^{-1}\right)-Carbon\;input\;\left(kg\;CE\;ha^{-1}\right)$$

(31) $$Carbon\;efficiency\;ratio=\frac{Carbon\;output\;\left(kg\;CE\;ha^{-1}\right)}{Carbon\;input\;\left(kg\;CE\;ha^{-1}\right)}$$

(32) $$Carbon\;efficiency\left(kg\;kg^{-1}\;CE\right)=\frac{Castor\;seed\;yield\;\left(kg\;ha^{-1}\right)}{Carbon\;input\;\left(kg\;CE\;ha^{-1}\right)}$$

(33) $$Carbon\;sustainability\;index=\frac{Net\;carbon\;gain\;\left(kg\;CE\;ha^{-1}\right)}{Carbon\;input\;\left(kg\;CE\;ha^{-1}\right)}$$

### 2.10 Statistical analysis

The observations recorded on the various growth, yield attributes, yield, N content in seed and stalk and also N uptake recorded during individual years (2022-2024) were averaged and tabulated. The experimental data were analyzed according to the standard methodology suggested by Gomez and Gomez (1984). The significance of the treatments was tested by the F test. The least significant differences between the treatment means were tested at a probability level of 0.05% by using the standard methodology suggested by Gomez and Gomez (1984). Similarly, various soil and plant-based N indices worked out based on the mean yield of three years. The treatment means subjected to one-way analysis of variance to test the significance of treatment effects at the 5% probability level. When significant differences among treatments were detected, treatment means were further compared using Duncan’s Multiple Range Test (DMRT) at *p* ≤ 0.05.

## 3 Result

### 3.1 Crop growth

The key growth attributes of castor varied significantly due to different N management treatments (**Table 1**). Irrespective of nutrient gradients crop has evidently benefited from the foliar application of N. Interestingly, foliar application of normal prilled urea showed a promising response over nano urea as a foliar nutrient in rainfed castor. Particularly, application of 100% RRBN through soil, followed by 2% urea spray at 30 and 60 DAS, showed significant (p ≤0.05) taller plants (55.4 cm), and more LAI (1.48 and 1.73, respectively, at 50 and 100 DAS). The SPAD chlorophyll meter reading at 20 DAS was highest with RRF (35.1), while it was significantly higher for 100% RRBN through soil, followed by 2% urea spray at 40 and 70 DAS. Due to taller plants and more leafiness, the total dry matter production (TDMP) was significantly (p ≤ 0.05) higher (92.4 and 202.7 g plant^− 1^ at 50 and 100 DAS, respectively) with 100% RRBN through soil, followed by 2% urea spray at 30 and 60 DAS. Similarly, applying 100% RRBN through soil, followed by 2% urea spray at 30 and 60 DAS, even outperformed other treatments in terms of yield-related traits. Significantly (p ≤ 0.05), a higher number of effective spikes (10.1), a longer primary spike (47.1 cm), an effective primary spike length (39.6 cm), a greater number of capsules on the primary spike (47.9), and a 100-seed weight (31.6 g) were observed with this treatment (**Table 2**).

**Table 1:** Effect of nitrogen management strategies on growth attributes of castor (Mean of 3 years).

Treatment	Plant height (cm)	Leaf Area Index		SPAD values			Number of nodes to the primary spike	Total dry matter production (g plant^− 1^)	
		50 DAS	100 DAS	20 DAS	40 DAS	70 DAS		50 DAS	100 DAS
RRF (40-40–25 kg N-P_2_O_5_-K_2_O ha^-1^)	46.6c	1.22b	1.37a-c	35.1b	38.1e	32.2g	12.0a	73.2c	174.4b
50% RRBN + 2 ml l^-1^ Nano urea at 30 and 60 DAS	42.5d	0.86c	1.12d	28.6f	37.8e	44.6f	12.7a	46.7f	123.8d
50% RRBN + 2% Urea spray at 30 and 60 DAS	47.9c	0.91c	1.18cd	28.1g	40.2d	48.4c	12.9a	48.1f	132.4d
75% RRBN + 2 ml l^-1^ Nano urea at 30 and 60 DAS	42.1d	1.31ab	1.51ac	31.2e	43.4c	46.7e	12.6a	60.6e	156.4c
75% RRBN + 2% Urea spray at 30 and 60 DAS	46.6c	1.34ab	1.62a	33.6d	46.7b	47.8d	12.7a	68.4d	160.5c
100% RRBN + 2 ml l^-1^ Nano urea at 30 and 60 DAS	51.0b	1.42a	1.68a	35.7a	46.3b	49.5b	12.7a	86.3b	197.9a
100% RRBN + 2% Urea spray at 30 and 60 DAS	55.4a	1.48a	1.73a	34.8c	54.6a	53.5a	13.0a	92.4a	202.7a
100% RRPK (00-40–25 kg N-P_2_O_5_-K_2_O ha^-1^)	23.6e	0.23d	0.19e	16.0g	12.5f	12.1h	12.1a	20.1g	27.4e
S. Em±	0.74	0.08	0.12	0.42	0.63	0.68	0.09	1.2	6.7
LSD (P = 0.05)	2.28	0.24	0.37	1.32	1.96	2.10	NS	3.7	20.04
CV (%)	17.15	10.8	14.75	16.7	23.4	14.23	12.27	10.5	14.4

RRF, Recommended rate of fertilizer; RRBN, Recommended rate of basal nitrogen; RRPK, Recommended rate of phosphorus and potassium, Mean followed by the same letter (s) within the column are not statistically differed by Duncan’s multiple range test (*p* ≤ 0.05).

**Table 2:** Effect of nitrogen management strategies on yield attributes of castor (Mean of 3 years).

Treatment	Number of effective spikes plant^-1^	Total primary spike length (cm)	Effective spike length of primary spike (cm)	No. of capsules per primary spike	100 seed weight (g)
RRF (40-40–25 kg N-P_2_O_5_-K_2_O ha^-1^)	8.3b	38.9bc	33.3b	38.1c	31.8b
50% RRBN + 2 ml l^-1^ Nano urea at 30 and 60 DAS	7.4c	33.2d	31.1c	31.1e	31.7b
50% RRBN + 2% Urea spray at 30 and 60 DAS	7.6c	35.7d	30.8cd	31.5e	31.8b
75% RRBN + 2 ml l^-1^ Nano urea at 30 and 60 DAS	7.6c	35.6d	29.1d	35.4d	32.0a
75% RRBN + 2% Urea spray at 30 and 60 DAS	8.4ab	36.9cd	31.1c	35.3d	31.5c
100% RRBN + 2 ml l^-1^ Nano urea at 30 and 60 DAS	8.7a	40.4b	33.9ab	40.8b	31.1d
100% RRBN + 2% Urea spray at 30 and 60 DAS	10.1a	47.1a	39.6a	47.9a	31.6ab
100% RRPK (00-40–25 kg N-P_2_O_5_-K_2_O ha^-1^)	0.3d	11.3e	9.7e	8.4f	24.1e
S. Em±	0.12	0.86	0.56	0.67	0.05
LSD (P = 0.05)	0.38	2.65	1.74	2.05	0.16
CV (%)	6.78	10.30	7.90	8.22	7.60

RRF, Recommended rate of fertilizer; RRBN, Recommended rate of basal nitrogen; RRPK, Recommended rate of phosphorus and potassium, Mean followed by the same letter (s) within the column are not statistically differed by Duncan’s multiple range test (*p* ≤ 0.05).

### 3.2 Yield and economics

Despite there was a notable variation in yield attributes seed yield of castor over the years of experimentation the productivity of castor showed a clear and significant variation across the N management practices (**Tables 2**, **3**). Castor responded positively to the applied N. Specifically, treatments with higher rates of N application recorded higher yields. Among the different treatments, application of 100% RRBN through soil, followed by foliar spray of 2% urea at 30 and 60 DAS recorded significantly (p ≤ 0.05) higher seed yield (1546 kg ha^-1^) and was remain on par with 100% RRBN + 2 ml l^-1^ Nano urea at 30 and 60 DAS (1402 kg ha^-1^), 75% RRBN + 2% urea spray at 30 and 60 DAS, soil application of RRF (1298 kg ha^-1^), 75% RRBN + 2% urea spray at 30 and 60 DAS (1262 kg ha^-1^). A significantly lower seed yield of castor (178 kg ha^−1^) was recorded under the 100% RRPK treatment. With regards to stalk yield treatment with higher rates of soil applied N (75-100% RRBN) followed by foliar application of N (urea and nano urea), were superior over soil application of RRF. Concerning biological yield treatments, the excelled superior option for seed and stalk yield surpassed others and was evident (**Table 3**).

**Table 3:** Effect of nitrogen management strategies on seed yield, stalk yield, and biological yield of castor.

Treatment	Seed yield (kg ha^-1^)				Stalk yield (kg ha^-1^)				Biological yield (kg ha-1)			
	2022	2023	2024	Pooled	2022	2023	2024	Pooled	2022	2023	2024	Pooled
RRF (40-40–25 kg N-P_2_O_5_-K_2_O ha^-1^)	1464bc	1043a	1387b-c	1298ab	2623bc	1909a	2621a-c	2384a-c	4087bc	2952a	4008ab	3682ab
50% RRBN + 2 ml l^-1^ Nano urea at 30 and 60 DAS	1217c	941b	1105c	1088b	2254c	1722a	2088c	2021c	3471c	2663a	3193c	3109c
50% RRBN + 2% Urea spray at 30 and 60 DAS	1341bc	895bc	1330b-d	1189b	2494bc	1638a	2514a-c	2215bc	3835c	2533a	3844bc	3404bc
75% RRBN + 2 ml l^-1^ Nano urea at 30 and 60 DAS	1257bc	972b	1223bc	1151b	2338c	1779a	2311bc	2143bc	3595c	2751a	3534bc	3293bc
75% RRBN + 2% Urea spray at 30 and 60 DAS	1379bc	1021a	1388b-d	1262ab	2565bc	1868a	2623a-c	2352a-c	3944c	2889a	4011ab	3615ab
100% RRBN + 2 ml l^-1^ Nano urea at 30 and 60 DAS	1585ab	1106a	1515cd	1402a	2948ab	2024a	2863ab	2612ab	4533ab	3130a	4378ab	4014ab
100% RRBN + 2% Urea spray at 30 and 60 DAS	1827a	1180a	1631d	1546a	3398a	2159a	3083a	2880a	5225a	3339a	4714a	4426a
100% RRPK (00-40–25 kg N-P_2_O_5_-K_2_O ha^-1^)	198d	165c	172a	178c	340d	323b	289d	317d	538d	488b	461d	496d
S. Em±	116	107	126	116	191	177	208	191	307	284	334	307
LSD (P = 0.05)	346	319	373	346	571	526	615	571	917	845	988	917
CV (%)	18	14	13	16	23	18	19	16	20	17	18	15

RRF, Recommended rate of fertilizer; RRBN, Recommended rate of basal nitrogen; RRPK, Recommended rate of phosphorus and potassium, Mean followed by the same letter (s) within the column are not statistically differed by Duncan’s multiple range test (*p* ≤ 0.05).

The oil content and oil yield were significantly influenced by N management (**Figure 2**). Increasing total N application rates progressively enhanced the oil content of castor seed, indicating a positive response of seed oil accumulation to improved N availability. Particularly, the seeds harvested in plots treated with 100% RRBN, followed by a 2% urea spray at 30 and 60 DAS, were found to be superior (42.7%) to those in over treatments. Whereas, the lowest oil content in the seed was observed with 100% RRPK, where N was omitted (33.8%). Oil yield, being a function of seed yield and oil content, closely followed the trends observed for both these parameters. Specifically, the treatment comprising 100% RRBN combined with a foliar application of 2% urea at 30 and 60 DAS yielded the highest oil content over other treatments (**Figure 3**).

**Figure 2:**
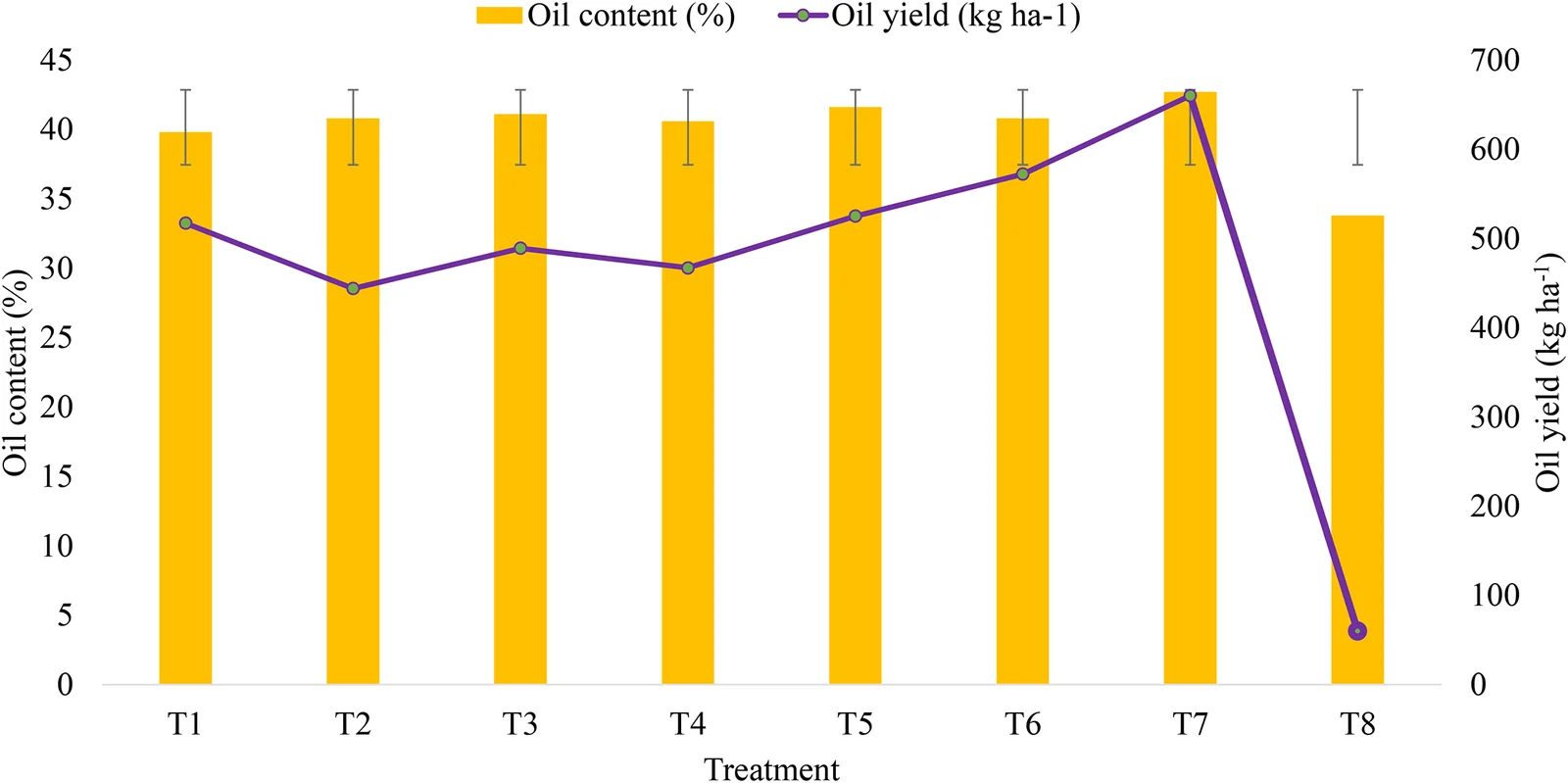
Effect of nitrogen management strategies on oil content and oil yield of castor (Mean of 3 years).

**Figure 3:**
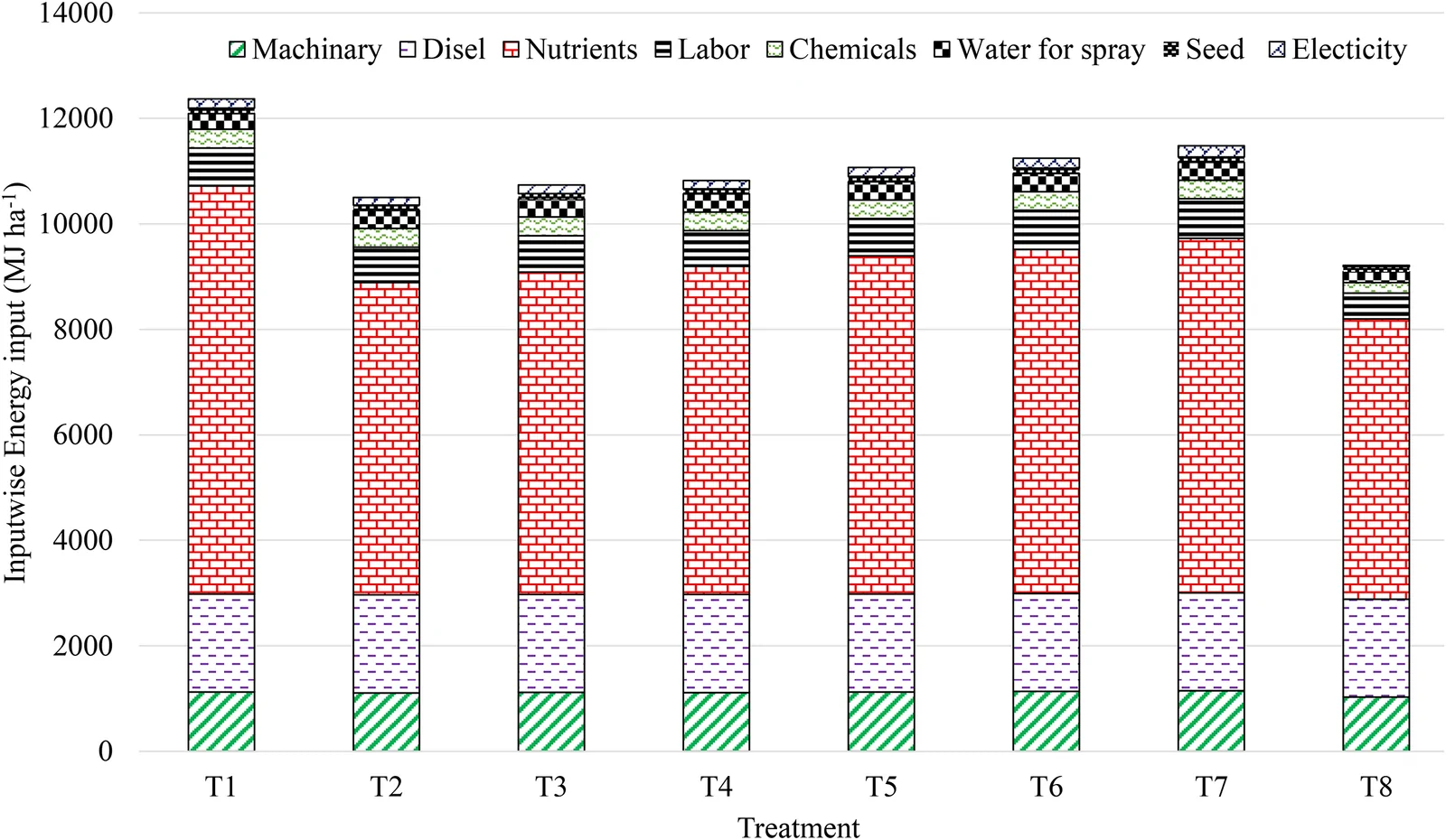
Contribution of different inputs to total energy input as influenced by nitrogen management in rainfed castor (Mean of 3 years).

The economic analysis of N management strategies was worked out and presented in **Table 4**. Despite being a costly practice, foliar nutrition appears to be a promising option for realizing higher returns. Application of 100% RRBN through soil, followed by foliar application of 2 ml L^−1^ nano urea, incurred a higher production cost (480 $ ha^−1^), which remained statistically comparable with most treatments except RRF (457 $ ha^−1^) and 100% RRPK (378 $ ha^−1^). While plots applied with 100% RRBN through soil, followed by foliar application of 2% urea, recorded higher gross returns (1174 $ ha^-1^) and were ~19% more profitable over RRF through soil. Similarly, in net returns, the former treatment excelled over others (704 $ ha^-1^), which was ~33% more remunerative than the conventional N management strategy (RRF). Due to higher gross returns coupled with a moderate cost of production, the treatment involving 100% RRBN applied through soil, followed by a foliar application of 2% urea, achieved the highest BCR of 2.50, surpassing all other treatments.

**Table 4:** Effect of nitrogen management strategies on the economics of castor production (Mean of 3 years).

Treatment	Coast of cultivation ($ ha^-1^)	Gross returns ($ ha^-1^)	Net returns ($ ha^-1^)	BCR
RRF (40-40–25 kg N-P_2_O_5_-K_2_O ha^-1^)	457	986	529	2.16
50% RRBN + 2 ml l^-1^ Nano urea at 30 and 60 DAS	477	826	350	1.73
50% RRBN + 2% Urea spray at 30 and 60 DAS	468	903	436	1.93
75% RRBN + 2 ml l^-1^ Nano urea at 30 and 60 DAS	478	874	396	1.83
75% RRBN + 2% Urea spray at 30 and 60 DAS	469	959	490	2.05
100% RRBN + 2 ml l^-1^ Nano urea at 30 and 60 DAS	480	1065	586	2.22
100% RRBN + 2% Urea spray at 30 and 60 DAS	470	1174	704	2.50
100% RRPK (00-40–25 kg N-P_2_O_5_-K_2_O ha^-1^)	378	135	-243	0.36

RRF, Recommended rate of fertilizer; RRBN, Recommended rate of basal nitrogen; RRPK, Recommended rate of phosphorus and potassium.

Market price of castor seed: 0.75 $ kg^-1.^

### 3.3 Nitrogen uptake and indices

**Table 5** presents the N content in the seed and stalk of the castor. The N content in plants varied with different management modules. Regardless of the soil applied N, foliar spray of urea enhanced N content in the seed. Specifically, application of 100% RRBN, followed by foliar spray of 2% urea at 30 and 60 DAS, recorded significantly (p ≤ 0.05) higher concentration of N (1.170%) than other treatments. Similar trends were also observed with the N content of the stalk, where 100% RRBN + 2% urea spray at 30 and 60 DAS recorded significantly higher N concentrations (0.784%). A significantly lower N content was observed in both seed and stalk with 100% RRPK (**Table 3**). The application of 100% RRBN, followed by a foliar spray of 2% urea at 30 and 60 DAS, resulted in higher N uptake by the seed (18.2 kg ha^-1^) compared to other treatments. It was even so with regards to N uptake by stalk (**Table 5**).

**Table 5:** Effect of nitrogen management strategies on nitrogen content and nitrogen uptake by seed and stalk of castor (Mean of 3 years).

Treatment	Nitrogen content in seed (%)	Nitrogen content in stalk (%)	Nitrogen uptake by seed (kg ha^−1^)	Nitrogen uptake by stalk (kg ha^−1^)	Total nutrient uptake (kg ha^−1^)
RRF (40-40–25 kg N-P_2_O_5_-K_2_O ha^-1^)	1.093b	0.732a-c	14.3a-c	12.9bc	27.2a
50% RRBN + 2 ml l^-1^ Nano urea at 30 and 60 DAS	0.995c	0.667c	10.9bc	10.0cd	20.9b
50% RRBN + 2% Urea spray at 30 and 60 DAS	1.047bc	0.701a-c	12.5bc	11.5cd	24.0b
75% RRBN + 2 ml l^-1^ Nano urea at 30 and 60 DAS	1.141ab	0.764a-c	13.2bc	12.1bc	25.3b
75% RRBN + 2% Urea spray at 30 and 60 DAS	1.155ab	0.774ab	14.6a-c	13.5a-c	28.1a
100% RRBN + 2 ml l^-1^ Nano urea at 30 and 60 DAS	1.145ab	0.767ab	16.1ab	14.8ab	30.9a
100% RRBN + 2% Urea spray at 30 and 60 DAS	1.170a	0.784a	18.2a	16.7a	34.9a
100% RRPK (00-40–25 kg N-P_2_O_5_-K_2_O ha^-1^)	0.865d	0.680bc	1.5d	1.6e	3.1c
S. Em±	0.02	0.03	1.34	1.22	4.20
LSD (P = 0.05)	0.07	0.10	4.12	3.76	13.4
CV (%)	7.34	6.65	8.73	8.41	10.22

RRF, Recommended rate of fertilizer; RRBN, Recommended rate of basal nitrogen; RRPK, Recommended rate of phosphorus and potassium, Mean followed by the same letter (s) within the column are not statistically differed by Duncan’s multiple range test (*p* ≤ 0.05).

Generally, N management strategies combining both soil and foliar applications resulted in higher PFP of N (**Table 6**). Regardless of N management strategies, the relationship between PFP and N rates was inversely proportional. Specifically, 50% RRBN + 2 ml l^-1^ nano urea at 30 and 60 DAS recorded the highest PFP of N (106.7 kg kg^−1^), closely followed by 50% RRBN, and then foliar spray of 2% urea at 30 and 60 DAS (92.9 kg kg^−1^). The lowest PFP of N (32.5 kg kg^−1^) was observed with soil application of RRF. The NBI values were positive in all treatments where integrated soil and foliar N applications were used.

**Table 6:** Effect of nitrogen management strategies on soil and plant nitrogen indices (Mean of 3 years).

Parameters	T1	T2	T3	T4	T5	T6	T7	T8
Partial factor productivity of nitrogen (kg kg^-1^)	32.5c	106.7a	92.9ab	75.7b	70.9b	69.4b	67.8b	–
Nitrogen balance intensity	-12.8g	10.7c	11.2b	10.1e	10.3d	10.7c	12.1a	3.1f
Nitrogen use efficiency of crop	0.68f	2.05a	1.88b	1.66c	1.58d	1.53e	1.53e	–
Agronomic efficiency	28.0g	89.2a	79.0b	64.0c	60.9d	60.6e	60.0f	–
Fertilizer nitrogen recovery efficiency	60.3g	174.5a	163.3b	146.1c	140.4d	137.6f	139.5e	–
Physiological efficiency	46.5c	51.1a	48.4b	43.8e	43.4f	44.0d	43.0g	–
Nitrogen utilization efficiency (kg kg^-1^)	47.7d	52.1b	49.5c	45.5e	44.9g	45.4f	44.3h	57.4a
Nitrogen use efficiency of soil	9.07d	8.26g	8.99e	8.64f	9.42c	10.39b	11.38a	1.36h
Nitrogen uptake efficiency (%)	6.70d	5.56g	6.34f	6.64e	7.32c	8.00b	8.98a	0.85h
Nitrogen use efficiency of yield	319.7d	289.2g	313.9e	301.9f	328.8c	363.0b	397.6a	48.6h

Mean followed by the same letter (s) within the rows are not statistically differed by Duncan’s multiple range test (*p* ≤ 0.05).

A higher NUE was observed at 50% RRBN and declined as the N rates were increased. A considerably lower NUE was observed under RRF, where only soil application of nutrients was used (**Table 6**). Similar trends were even observed with AE and FNRE, where 50% RRBN through soil followed by 2 ml l^-1^ nano urea at 30 and 60 DAS (89.2 and 174.5%, respectively) or 2% urea spray at 30 and 60 DAS (79.0 and 163.3%, respectively) recorded significantly higher AE and FNRE, respectively. The PE values were relatively stable across the treatments, ranging from 43.0 to 51.1, as usual, lower rates of N application outperformed over elevated levels of N. The NUtE was usually more under N omitted plots and was so happened with RRPK (57.4 kg kg^-1^), while it was minimum in 100% RRBN + 2% urea spray at 30 and 60 DAS (44.3 kg kg^-1^) and slightly enhanced with the successive lower rates of N application (**Table 6**). In contrast, reverse trends were observed with NUES and NUtE (**Table 6**), where higher rates of N showed higher values of NUES and NUtE. These were notably higher under 100% RRBN + 2% urea spray at 30 and 60 DAS. Likewise, the NUEY values indicated that 100% RRBN + 2% urea spray at 30 and 60 DAS was found exceptionally superior among the different treatments (397.6) and declined as the fertilizer rates were reduced from 100% RRBN to 50% RRBN. However, RRF showed a comparable response to that of 75% RRBN, followed by urea or nano-urea sprays (**Table 6**).

### 3.4 Energy analysis

The energy metrics of different N management modules are summarized in **Table 7**. Soil application of RRF was found to be an energy-intensive strategy when compared to other (12370 MJ ha^-1^); however, it was closely followed by 100% RRBN, followed by 2% urea (11481 MJ ha^-1^) or 2 ml l^-1^ nano urea (11246 MJ ha^-1^) spray at 30 and 60 DAS. Treatment with no N application (RRPK) showed the lowest energy input (9216 MJ ha^-1^). Like energy input, energy output was also varied significantly among the treatments. A higher energy output was observed with the application of 100% RRBN through the soil, followed by a foliar spray of 2% urea at 30 and 60 DAS (58726 MJ ha^-1^). It was again comparable with that of 100% RRBN through soil, followed by foliar spray of 2 ml l^-1^ nano urea at 30 and 60 DAS (53259 MJ ha^-1^). The energy output of these treatments was ~20% and 8% higher than the conventional soil-based application of RRF (48881 MJ ha^-1^). The highest energy efficiency was observed with the application of 100% RRBN, followed by 2% urea or 2 mL l^-1^ nano urea spray at 30 and 60 DAS, attributed to the highest net energy gain (**Table 7**). Furthermore, energy profitability, energy productivity, and nutrient energy ratio were also remarkably higher with 100% RRBN + 2% urea spray at 30 and 60 DAS (4.11, 0.39, and 8.74). Direct energy consumption remained stable across the treatments (2681–2820 MJ ha^-1^), except in RRPK (**Table 7**). Treatment showed considerable variation in indirect energy usage. Significantly higher indirect energy input was observed in 100% RRBN + 2% urea spray at 30 and 60 DAS (55907 MJ ha-1); this was comparable to 100% RRBN + 2 ml l^-1^ nano urea at 30 and 60 DAS (50469 MJ ha^-1^). Conversely, non-renewable energy input was significantly higher with soil application of RRF (6651 MJ ha^-1^); otherwise, it was stable across the other treatments except RRPK (**Table 7**). Interestingly, specific energy was notably highest in RRPK (18.58 MJ ha^-1^) than in other treatments.

**Table 7:** Effect of nitrogen management strategies on energy flow in castor (Mean of 3 years).

Parameters	T1	T2	T3	T4	T5	T6	T7	T8
Energy input (MJ ha^-1^)	12370a	10504g	10735f	10825e	11070d	11246c	11481b	9216h
Energy output (MJ ha^-1^)	48881c	41256g	45166e	43707f	47951d	53259b	58726a	6579h
Energy efficiency	3.95f	3.93g	4.21d	4.04e	4.33c	4.74b	5.11a	0.71h
Net energy gain (MJ ha^-1^)	36510d	30752g	34431e	32883f	36881c	42013b	47245a	-2637h
Energy profitability	2.95f	2.93g	3.21d	3.04e	3.33c	3.74b	4.11a	-0.29h
Energy productivity	0.30e	0.30e	0.32d	0.30e	0.33c	0.36b	0.39a	0.05f
Direct energy (MJ ha^-1^)	2756c	2681g	2710e	2693f	2736d	2790b	2820a	2371h
Indirect energy (MJ ha^-1^)	46124c	38575g	42456e	41014f	45216d	50469b	55907a	4209h
Renewable energy (MJ ha^-1^)	5719e	5714g	5730d	5718f	5746c	5781b	5791a	5392h
Non-renewable energy (MJ ha^-1^)	6651a	4789g	5005f	5106e	5324d	5465c	5691b	3824h
Specific energy (MJ ha^-1^)	3.36c	3.38b	3.15e	3.29d	3.06f	2.80g	2.59h	18.58a
Nutrient energy ratio	6.32g	6.97f	7.39d	7.02e	7.47c	8.16b	8.74a	1.24h

Mean followed by the same letter (s) within the rows are not statistically differed by Duncan’s multiple range test (*p* ≤ 0.05).

### 3.5 Carbon auditing

The study revealed significant variations in carbon footprints across different treatments (**Table 8**). The highest C input was observed with conventional soil application of RRF (317 kg CE ha^−1^) compared to other treatments, while it was lowest in RRPK (216 kg CE ha^−1^). Conversely, C output was exceptionally high with 100% RRBN + 2% urea spray at 30 and 60 DAS (1947 kg CE ha^−1^) relative to other treatments. Interestingly, higher C footprint values were associated with the application of RRPK (1.22 kg CE kg^−1^ castor yield). Furthermore, treatments involving 50-75% RRBN with foliar fertilization of urea and/or nano urea and soil application of RRF showed higher C footprints than 100% RRBN, followed by 2% urea spray or 2 ml l^-1^ nano urea at 30 and 60 DAS (**Table 8**). Due to their higher C efficiency ratio and overall C efficiency, the application of 100% RRBN, followed by 2% urea spray (1642 kg CE ha^−1^) and/or 2 ml l^-1^ nano urea (1467 kg CE ha^−1^) at 30 and 60 DAS demonstrated the highest net C gain. Finally, the C sustainability index was also highest with these treatments, and was significantly lowest in RRPK (0.01).

**Table 8:** Effect of nitrogen management strategies on carbon footprints of castor (Mean of 3 years).

Parameters	T1	T2	T3	T4	T5	T6	T7	T8
Carbon input (kg CE ha^−1^)	317a	275f	282e	282e	291d	299c	306b	216g
Carbon output (kg CE ha^−1^)	1620c	1368g	1498e	1449f	1590d	1766b	1947a	218h
Carbon Footprinting (kg CE kg^− 1^ castor yield)	0.24d	0.25e	0.24d	0.25e	0.23c	0.21b	0.20a	1.22f
Net carbon gain (kg CE ha^−1^)	1303c	1093g	1216e	1167f	1299d	1467b	1642a	1.34h
Carbon efficiency ratio	5.11f	4.98g	5.31d	5.13e	5.46c	5.91b	6.37a	1.01h
Carbon efficiency (kg kg^− 1^ CE)	4.09e	3.96g	4.22d	4.08f	4.34c	4.69b	5.05a	0.82h
Carbon sustainability index	4.11f	3.98g	4.31d	4.13e	4.46c	4.91b	5.37a	0.01h

Mean followed by the same letter (s) within the rows are not statistically differed by Duncan’s multiple range test (*p* ≤ 0.05).

## 4 Discussion

### 4.1 Weather and crop performance

The rainfall is a vital determinant of crop productivity in rainfed regions (Thimmegowda et al., 2025). Although castor is a drought-tolerant crop, moisture stress at critical growth stages affects its performance (Kumar and Yamanura, 2019). Rainfall is the primary driver of balanced nutrition; dry spells during the growing season hinder nutrient management practices. Likewise, we observed a marked variation in crop productivity over the years (**Tables 2**, **3**). However, treatment effects were consistent over the seasons. The growth and yield of the crop during 2022–23 and 2023–24 were superior to those in 2024-25. The seasonal variation in productivity was purely due to variation in rainfall. The rainfall received during 2022–23 and 2024–25 was nearly 109 and 101% higher than 2023-24, respectively (**Figure 1**). The suboptimal and unevenly distributed rainfall during 2023–24 led to prolonged moisture deficits in the cropping period, particularly in the early vegetative stage (August), at primary spike initiation (October), and extending into the late reproductive phase. As a result, crop growth and performance in the former years were superior to those in the latter years across all treatments. In 2022–23 and 2024-25, most of the rainfall received coincided with the critical growth stages, resulting in crops enjoying congenial weather, which contributed to better growth and productivity. This result aligns with the findings of Kumar et al. (2021), who observed the superior performance of various castor hybrids and varieties under high and well-distributed rainfall regimes.

### 4.2 Crop growth

Under identical soil and environmental conditions, variations in nutrient application result in differential crop performance (Reddy et al., 2024; Patel et al., 2023). Similarly, in this study, key growth attributes of castor, such as plant height, LAI, chlorophyll status of leaves, and TDMP at different growth phases, were consistently superior with the application of 100% RRBN through soil, followed by 2% urea spray at 30 and 60 DAS (**Table 1**). This could be due to the better nourishment of the crop through combined soil and foliar modes of nutrient application at relatively higher rates. In the combined supply of N, soil-applied N contributed to the initial establishment of the crop, followed by foliar application of N through urea, which may have directly reached the site of action. Evidently, foliar application of N recorded significantly higher SPAD values at 40 and 60 DAS when compared with conventional soil-applied nitrogen (RRF) may be attributed to the rapid and direct absorption of N through the leaf surface might have facilitated the efficient incorporation of N into chlorophyll which improved leaf N status likely enhanced chlorophyll synthesis and photosynthetic activity, thereby increasing assimilate production and translocation to reproductive sinks, which ultimately contributed to better growth and yield-attributing traits in castor (Kumar et al., 2023). In contrast, the comparatively lower SPAD values under conventional soil application of N may be associated with the inconsistent soil-moisture regime resulted due to uneven distribution of rainfall, coupled with fewer rainy days during the crop season (**Figure 1**), which could have restricted N dissolution, diffusion and root uptake. Integration of foliar nutrition in nutrient management of castor makes timely provision of optimal nutrient levels facilitated plant metabolism and a range of physiological processes, including cell division, chlorophyll biosynthesis, and enzyme activation, collectively contributing to enhanced crop growth and development (Kumar et al., 2024). Despite higher rates of N application in RRF, suboptimal performance of the crop could be due to lower N use efficiency of soil-applied N under rainfed situations (Congreves et al., 2021).

According to Rowlings et al. (2022), the low N use efficiency and suboptimal crop performance under rainfed conditions are attributed to moisture deficits, asynchronous nutrient availability, and multiple pathways of N loss. Furthermore, the lower efficacy of nano urea compared to normal prilled urea may be due to a thick cuticular wax layer on the castor leaves, which inherently restricts the entry of the nutrient solution. The presence of a hydrophobic barrier often restricts the efficacy of foliar-applied nutrient solutions in castor. Particularly, nano urea, containing a relatively lower N content (~4%) compared to prilled urea (46%), might be less effective in achieving the desired agronomic outcomes. Similar opinions were earlier reported by Upadhyay et al. (2023). Despite this issue, the promising response of normal urea solution sprayed at a higher concentration (2%) might have facilitated greater uptake by the plant, thereby enhancing N assimilation and promoting growth in castor.

### 4.3 Yield attributes

The yield attributes are the manifestation of growth parameters. According to earlier studies by Kumar et al. (2023); Kumar et al. (2024), and Kumar et al. (2025), a high degree of positive correlation was observed between growth and yield attributing traits in rainfed castor. Better growth treatment with the application of 100% RRBN through the soil, followed by a 2% urea spray at 30 and 60 DAS, showed higher values for the number of effective spikes, longer primary spike length, effective primary spike length, and a greater number of primary spikes, as well as a higher 100-seed weight. In the said treatment, along with the good growth foliar feeding of N at 30 and 60 DAS, perfectly coincided with the primary, secondary, and third-order spike induction stage. These spikes in rainfed castor nearly contribute to 80-95% of the yield (Yamanura et al., 2022). The synchronized supply of N at relatively higher rates in the former treatment led to optimal photosynthesis and its partitioning, resulting in better yield attributes. For instance, the higher units of yield attributed to traits with a foliar supplement of water-soluble complex fertilizer were earlier reported by Ramanjaneyulu et al. (2021) and Kumar et al. (2024).

### 4.4 Seed yield and oil yield

The seed yield is a key indicator of yield-related traits and which in term connected with growth and development of crop. In this study we observed a marked variation in productivity of castor over the years (**Table 3**) and was closely associated with rainfall and its distribution. Higher crop productivity during 2022–23 and 2024–25 was attributed to greater and well-distributed rainfall (1491 and 1439 mm, respectively) received over 64 rainy days, compared with 712 mm over 44 rainy days in 2023–24 (**Figure 1**). The lower and erratic rainfall of 2023–24 likely induced moisture stress which restricted crop growth and reduced yield across treatments. However, seed yield of castor showed a clear trend due to N management practices across the years of investigation. Application of 100% RRBN through soil, followed by a foliar spray of 2% urea at 30 and 60 DAS showed higher productivity over other treatments. This could be due to development of higher units of growth and yield attribution traits (**Tables 1**, **2**). These results support the idea of partial supplementation of the recommended N and underscore the importance of foliar nutrition in nutrient management of climatically challenged crop production systems. In addition to seed yield, stalk yield was also improved with foliar N application, especially with 2% urea at 30 and 60 DAS. Numerous experiments on various rainfed crops have consistently demonstrated the beneficial effects of foliar urea application (Ramanjaneyulu et al., 2021; Kumar et al., 2024). Combining soil and foliar application of urea works synergistically to enhance yield effectively. Soil-applied N supports fundamental plant growth by nourishing the root zone, while foliar N feeding during critical growth stages quickly supplies N directly to the leaves. This practice is especially important in rainfed, moisture-limited conditions where nutrient uptake through roots may be restricted.

Optimal nutrient management can significantly boost oil content. Similarly, seeds harvested from plots where 100% RRBN was used, along with a 2% urea spray at 30 and 60 DAS, showed higher oil content (42.7%) compared to others. Nitrogen is a vital nutrient; its optimal supply primarily promotes the uptake of other essential nutrients from the soil, especially sulfur (Mukhtar et al., 2022), which is crucial for the biosynthesis of fatty acids. Many studies have shown the close relationship between N and sulfur in the plant system (Mukhtar et al., 2022; Talukdar et al., 2022). The combined application of N through soil and foliar methods may have collectively played a key role in overall growth and development, as well as in catalyzing the essential metabolic reactions needed for oil biosynthesis in castor. Furthermore, oil yield was also higher with this treatment, likely due to increased yield coupled with higher oil content. Higher oil yield, along with higher seed yield and oil content, has been previously reported by Ramanjaneyulu et al. (2021); Kumar et al. (2023); Kumar et al. (2024); and Kumar et al. (2025).

### 4.5 Economics

Profitability is a key concern in the production of non-edible crops. Castor, being a market-oriented crop, the profit earned per rupee invested determines its viability. The integration of foliar nutrition practices increased the cost of castor production due to additional investments in labor and equipment, which are costly inputs. This could be due to the additional labor required for foliar fertilizer application, as well as the extra units of labor, machinery, and equipment used in harvesting and processing. The slightly higher cost of cultivation (1.96%) under 100% RRBN + 2 mL L^−1^ nano urea at 30 and 60 DAS, when compared with 100% RRBN + 2% urea spray at the same stages, could primarily be attributed to the relatively higher cost of nano urea (₹ 0.45 ml^-1^) than conventional urea (₹ 0.00592 g^-1^). Furthermore, analysis of gross and net returns showed that application of 100% RRBN through soil, followed by foliar application of 2% urea, recorded higher gross returns (~19%) and net returns (~33%) compared to the conventional RRF, due to the significantly higher yield from the former treatment (**Tables 3**, **4**). Additionally, due to higher gross returns and moderate production costs, 100% RRBN applied through soil, followed by foliar application of 2% urea, achieved a higher BCR (2.50) than other treatments. These results align with the findings of Kumar et al. (2023).

### 4.6 Nitrogen content and uptake

Plant nutrients fuel multiple physiological processes in crop plants, starting from root development to flowering and seed setting. In general, effective nutrient absorption maximizes the yield and quality of the produce. Assessment on nutrient content of seed and stalk of the castor plant in different treatment variants showed that integrated supply of N through soil and foliar means, especially when provided at higher rates, recorded higher N content in the castor seed and stalk (**Table 5**). Higher rates of N available in the root zone are followed by relatively higher concentrations of nutrient solution supplied through the leaves triggers a rapid acceleration of nitrogen assimilation machinery in plants, which rapidly increase nitrate reductase activity, amino acid synthesis via glutamine synthetase pathway as a result it triggers the overall growth and physiological functions of the crop (Ali, 2020). Therefore, optimal N absorption took which eventually encourage nutrient uptake. Higher rates of nutrient uptake under combined supply of soil and foliar fertilization was earlier reported by Kumar et al. (2023) and Gato et al. (2024).

The impact of varied N supply was further elucidated through detailed analysis of plant and soil-based N indices, revealing distinct patterns in N dynamics and crop responsiveness. The N levels and PFP established a perfect inverse relationship validated the phenomenon of diminishing marginal response, wherein increasing N supply beyond optimal levels reduced efficiency of its utilization (**Table 6**). According to IPNI (2014), a positive value of NBI likely reflects a decline in soil fertility. This study showed that the positive value of NBI across integrated soil cum foliar N management treatments reflecting a net depletion of soil N reserves, suggesting inadequate replenishment and a gradual decline in soil fertility. The consequences of N depletion in the soil under foliar based nutrient management practices was again observed with the higher values of NUE_Crop_ (**Table 6**). The NUE_Crop_ value of 0.68 in the RRF treatment pointed to N surplus soil system, while values exceeding unity in other treatments denoted net N removal from the soil (Martinez-Feria et al., 2018). The AE and FNRE followed a trend similar to PFP, highlighting differential crop responsiveness to applied N (Dobermann, 2007). The PE of N management strategies reflect the capacity of the crop to utilize absorbed N for biomass production and yield formation. In the present study, PE remained relatively stable across N management treatments, ranging from 43.0 to 51.1 kg kg^−1^. This consistency suggests that, despite variations in soil-applied N levels and foliar N supplementation, the crop efficiently converted absorbed N into economic yield. Higher NUtE in N-omitted plots, particularly under RRPK (57.4 kg kg^−1^), demonstrated the soil’s inherent N-supplying capacity and the diminishing performance under excessive N inputs. The highest NUtE (44.3 kg kg^−1^) under 100% RRBN + 2% urea spray at 30 and 60 DAS confirmed the pronounced N responsiveness in castor under optimal supply conditions. The inverse association between NUE_Yield_ and NUtE further indicated efficient soil N contribution toward yield formation under combined soil and foliar N supplementation.

### 4.7 Energy analysis

Nutrient management is an energy-intensive practice. Many studies in the past have greatly emphasized the energy aspects of nutrient management (Parihar et al., 2018; Kumar et al., 2023, 2024; Gopinath et al., 2025; Kumar et al., 2025). Concerning energy input, soil-based N management (RRF) was exceptionally energy-intensive (12370 MJ ha^-1^) due to ~50% extra N used in fertilization (**Supplementary Table 1**). N is the energy-intensive input used in crop production; its usage in every additional unit escalates the energy input (**Figure 3**). Regardless of the nutrient management modules, nutrition surpassed the other inputs and practices in terms of total energy input (**Figure 3**). The greater share of nutrients in the total energy input of castor production ascribed to elevated N usage was earlier highlighted by Kumar et al. (2025). Despite higher energy usage in harvesting and post-harvest processing, the application of 100% RRBN, followed by urea and nano urea spray at 30 and 60 DAS, resulted in ~7% and 9% lower energy input, respectively. Conversely, 100% RRBN through soil, followed by foliar spray of 2% urea at 30 and 60 DAS, showed substantially higher energy output (58726 MJ ha^-1^). It was ~20% higher than the conventional soil-based application of RRF (48881 MJ ha^-1^). This is mainly due to the higher biomass generated in the former treatment. Furthermore, the higher energy output of the said treatment was attributed to its higher net energy gain, energy efficiency, energy profitability, energy productivity, and nutrient energy ratio (**Table 7**). Parihar et al. (2018) suggested that higher energy productivity and energy profitability lead to increased energy gain in various crops and cropping systems. Direct energy consumption remained stable across the treatments (2681–2820 MJ ha^-1^), except in RRPK (**Table 7**). Treatment showed considerable variation in indirect energy usage. Significantly higher indirect energy was observed in 100% RRBN + 2% urea spray at 30 and 60 DAS (5,5907 MJ ha^-1^) due to the additional hours of machinery (decorticator) used to achieve enhanced yield levels. The non-renewable energy input was significantly higher with soil application of RRF (6651 MJ ha^-1^) due to its N expense (**Supplementary Table 1**).

### 4.8 Carbon auditing

Rising CO_2_ levels in the atmosphere are raising concerns for life on Earth. Unfortunately, agriculture is often viewed as a significant source of greenhouse gases. N is a major contributor to GHG emissions. Therefore, its management in crop production should be reviewed from an environmental perspective. In this study, conventional soil-based N application (RRF) resulted in the highest C input (317 kg CE ha^−1^), primarily due to higher N application rates (**Supplementary Table 1**; **Figure 4**). N is the one input with the greatest potential for C emissions (1.3 kg CE kg^-1^); overuse would increase C emissions in the production system. Interestingly, C input from FYM was significantly higher in the treatment that included foliar N supplementation. Otherwise, N supplied through chemical fertilizers was a notable contributor to C input in castor production (**Figure 4**). Conversely, the application of 100% RRBN + 2% urea spray at 30 and 60 DAS resulted in the highest C output (1947 kg CE ha^−1^), net carbon gain (kg CE ha^−1^), C efficiency ratio, C efficiency (kg kg^-1^ CE), and C sustainability index (**Table 8**). This is likely due to higher C output driven by increased biological productivity (**Table 8**). The combined use of soil and foliar fertilization effectively supported balanced crop nutrition. After an initial application of higher rates of soil fertilizer, consistent nutrient availability through foliar feeding during key growth stages, using a 2% urea spray at 30 and 60 DAS, significantly helped the crop produce higher seed and stalk yields. The connection between higher biological yield and increased C output aligns with previous research by Kumar et al. (2023).

**Figure 4:**
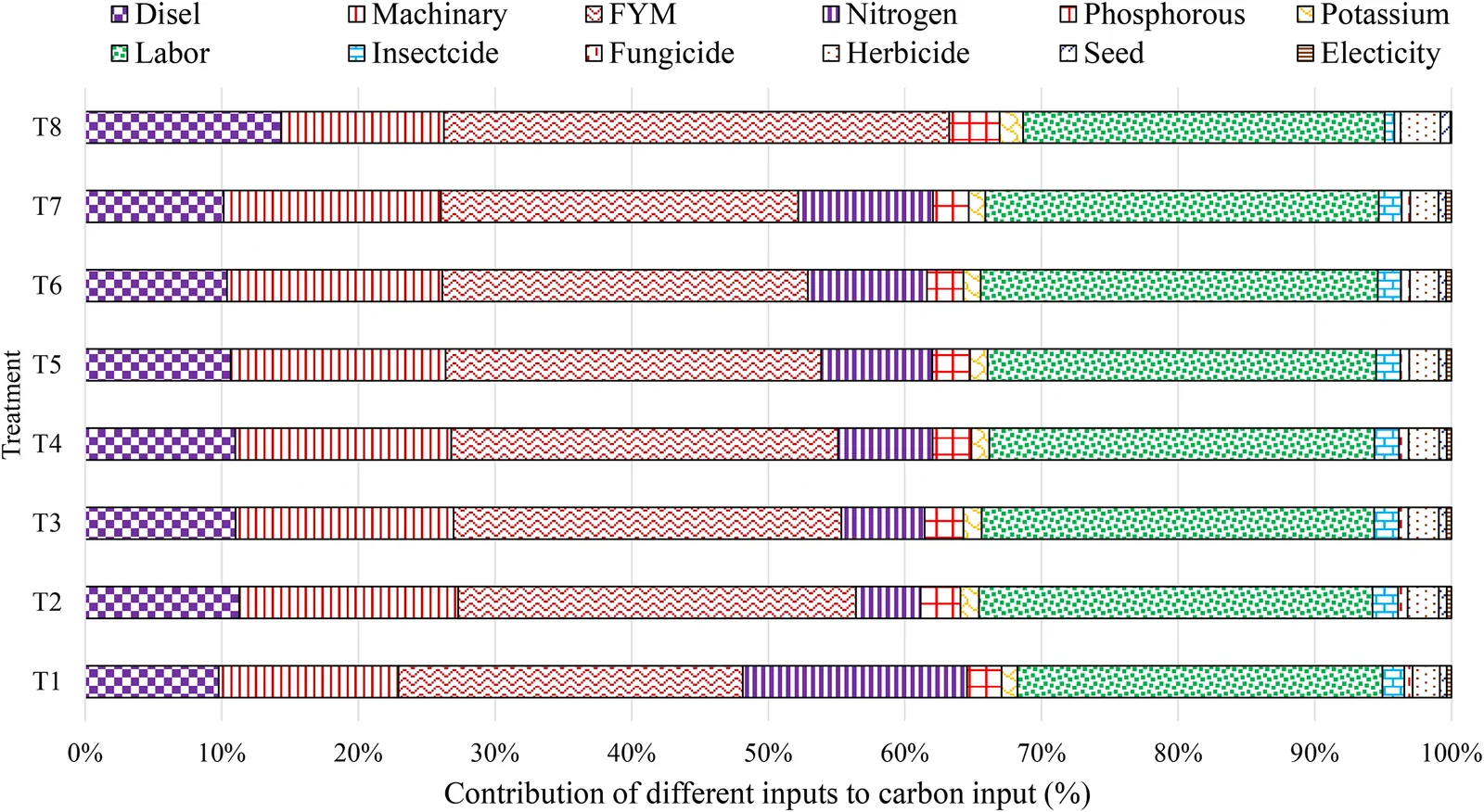
Percent contribution of different inputs to total carbon input as influenced by nitrogen management in rainfed castor (Mean of 3 years).

## 5 Conclusion

The study clearly shows that N management has a crucial impact on performance of rainfed castor. Among various N management strategies, the integrated soil and foliar application proved to be more effective in enhancing growth and yield traits when compared to conventional soil-based N application. Specifically, applying a 100% recommended rate of basal N through the soil, followed by foliar spray of 2% urea at 30 and 60 DAS enhanced growth and yield physiognomies of rainfed castor. This approach resulted in the highest seed yield, oil yield, as well as maximum net returns and benefit-cost ratio. N uptake, use efficiency, and energy metrics further confirmed the benefits of integrated N management, showing higher N content in seeds and stalks, improved N uptake, and increased energy efficiency. Additionally, C footprint analysis indicated that combined soil and foliar N application not only maximized C output but also achieved optimal C efficiency and sustainability than RRF and N omission treatments. Conversely, partial substitution of N demand of crop through foliar means depletes soil N as it was indicated by recording +ve N balance intensity (3-12.1) when compare to soil application of RRF (-12.8). Nevertheless, integrated soil cum foliar fertilization of N is essential for enhancing productivity, resource use efficiency, and environmental sustainability in rainfed castor cultivation.

## Data Availability

The original contributions presented in the study are included in the article/**Supplementary Material**. Further inquiries can be directed to the corresponding author.

## Supplementary material

Supplementary Table 1 Fertilization schedule of the study.

Supplementary Table 2 The energy equivalent of agricultural operations used in this study as both inputs and outputs.

Supplementary Table 3 The carbon equivalent of agricultural operations used in this study.
