# Supplementary material for: Nitrogen management in rainfed castor: a comprehensive analysis of productivity, nutrient use efficiency, economics, energy flow, and carbon footprint
Source: Front Plant Sci. 2026 Oct 28;17:1969600. doi: 10.3389/fpls.2026.1969600 (PMC13751998; doi:10.3389/fpls.2026.1969600)
Supplement: Supplementary file 1 [file Table1.docx]

**Supplementary Table 1. Fertilization schedule of the study**

| Treatment | Showing (kg ha^-1^) | | | 30 DAS (kg ha^-1^) | | | 60 DAS (kg ha^-1^) | | | Total (kg ha^-1^) | | |
| --- | --- | --- | --- | --- | --- | --- | --- | --- | --- | --- | --- | --- |
|  | N | P | K | N | P | K | N | P | K | N | P | K |
| RRF (40-40-25 kg N-P_2_O_5_-K_2_O ha^-1^) | 20.0 | 40.0 | 20.0 | 10.0 | 0.0 | 0.0 | 10.0 | 0.0 | 0.0 | 40.0 | 40.0 | 20.0 |
| 50% RRBN (20-40-25 kg N-P_2_O_5_-K_2_O ha^-1^) + 2 ml l^-1^ Nano urea at 30 and 60 DAS | 10.0 | 40.0 | 20.0 | 0.1 | 0.0 | 0.0 | 0.2 | 0.0 | 0.0 | 10.2 | 40.0 | 20.0 |
| 50% RRBN (20-40-25 kg N-P_2_O_5_-K_2_O ha^-1^) + 2% Urea spray at 30 and 60 DAS | 10.0 | 40.0 | 20.0 | 0.9 | 0.0 | 0.0 | 1.8 | 0.0 | 0.0 | 12.8 | 40.0 | 20.0 |
| 75% RRBN (30-40-25 kg N-P_2_O_5_-K_2_O ha^-1^) + 2 ml l^-1^ Nano urea at 30 and 60 DAS | 15.0 | 40.0 | 20.0 | 0.1 | 0.0 | 0.0 | 0.2 | 0.0 | 0.0 | 15.2 | 40.0 | 20.0 |
| 75% RRBN (30-40-25 kg N-P_2_O_5_-K_2_O ha^-1^) + 2% Urea spray at 30 and 60 DAS | 15.0 | 40.0 | 20.0 | 0.9 | 0.0 | 0.0 | 1.8 | 0.0 | 0.0 | 17.8 | 40.0 | 20.0 |
| 100% RRBN (40-40-25 kg N-P_2_O_5_-K_2_O ha^-1^) + 2 ml l^-1^ Nano urea at 30 and 60 DAS | 20.0 | 40.0 | 20.0 | 0.1 | 0.0 | 0.0 | 0.2 | 0.0 | 0.0 | 20.2 | 40.0 | 20.0 |
| 100% RRBN (40-40-25 kg N-P_2_O_5_-K_2_O ha^-1^) + 2% Urea spray at 30 and 60 DAS | 20.0 | 40.0 | 20.0 | 0.9 | 0.0 | 0.0 | 1.8 | 0.0 | 0.0 | 22.8 | 40.0 | 20.0 |
| 100% RRPK (00-40-25 kg N-P_2_O_5_-K_2_O ha^-1^) | 0.0 | 40.0 | 20.0 | 0.0 | 0.0 | 0.0 | 0.0 | 0.0 | 0.0 | 0.0 | 40.0 | 20.0 |
