# Supplementary material for: Nitrogen management in rainfed castor: a comprehensive analysis of productivity, nutrient use efficiency, economics, energy flow, and carbon footprint
Source: Front Plant Sci. 2026 Oct 28;17:1969600. doi: 10.3389/fpls.2026.1969600 (PMC13751998; doi:10.3389/fpls.2026.1969600)
Supplement: Supplementary file 2 [file Table2.docx]

**Supplementary Table 2.** The energy equivalent of agricultural operations used in this study as both inputs and outputs

| Inputs | Unit | Energy coefficient (MJ) | Reference |
| --- | --- | --- | --- |
| Tractor 50 HP | kg | 64.8 | Parihar et al. (2018) |
| Plough | kg | 62.70 | Parihar et al. (2018) |
| Tooth cultivator | kg | 22.80 | Dagistan et al. (2009) |
| Rotovator | kg | 6.69 | Kitani (1999) |
| Sprayer | kg | 0.17 | Kitani (1999) |
| Thresher | kg | 68.4 | Nassiri and Singh (2009) |
| Sickle | kg | 0.836 | Nassiri and Singh (2009) |
| Diesel | l | 56.31 | Parihar et al. (2018) |
| Labor | h | 1.96 | Parihar et al. (2018) |
| FYM | kg | 0.47 | Parihar et al. (2018) |
| Nitrogen | kg | 60.60 | Parihar et al. (2018) |
| Phosphorous | kg | 11.1 | Parihar et al. (2018) |
| Potassium | kg | 6.70 | Parihar et al. (2018) |
| Seed | kg | 20.4 | Campos et al. (1998) |
| Pendimethalin | kg | 150.9 | Green (1987) |
| Profenofos | kg | 184.63 | Parihar et al. (2018) |
| Propiconazole | kg | 97 | Parihar et al. (2018) |
| Water for spray | M3 | 0.17 | Mandal et al. (2002) |
| Output |  |  |  |
| Seed | kg | 14.7 | Mandal et al. (2002) |
| Stalk | kg | 12.5 | Mandal et al. (2002) |

h: Hours kg: kilogram, l: Liter, M^3^: meter cube
