# Supplementary material for: Nitrogen management in rainfed castor: a comprehensive analysis of productivity, nutrient use efficiency, economics, energy flow, and carbon footprint
Source: Front Plant Sci. 2026 Oct 28;17:1969600. doi: 10.3389/fpls.2026.1969600 (PMC13751998; doi:10.3389/fpls.2026.1969600)
Supplement: Supplementary file 3 [file Table3.docx]

**Supplementary Table 3.** The carbon equivalent of agricultural operations used in this study

| Inputs | Unit | Coefficient | References |
| --- | --- | --- | --- |
| Machinery | hr | 0.89 | Lal (2004) |
| Diesel | l | 0.94 | Lal (2004) |
| Labor | hr | 0.23 | Lal (2004) |
| FYM | kg | 0.008 | Lal (2004) |
| Nitrogen | kg | 1.3 | Lal (2004) |
| Phosphorous | kg | 0.2 | Chaudhary et al. (2017) |
| Potassium | kg | 0.15 | Lal et al. (2019) |
| Seed | kg | 0.32 | Wang et al. (2015) |
| Insecticide | kg | 5.1 | Lal (2004) |
| Fungicide | kg | 3.9 | Lal (2004) |
| Herbicide | kg | 6.3 | Lal (2004) |
| Electricity | kWh | 0.0725 | Lal (2004) |
| Biomass | kg | 0.44 | Lal (2004) |

h: Hours kg: kilogram, l: Liter, kWh: kilowatt-hour
